# Hind Right Approach Pancreaticoduodenectomy: From Skill to Indications

**DOI:** 10.1155/2014/210835

**Published:** 2014-08-10

**Authors:** Stefan Georgescu, Corina Ursulescu, Valentin Titus Grigorean, Cristian Lupascu

**Affiliations:** ^1^Department of Surgery, “Gr. T. Popa” University of Medicine and Pharmacy Iaşi, University Hospital “St. Spiridon” Iaşi, 1 Bulevardul Independentei, 700111 Iaşi, Romania; ^2^Department of Radiology, “Gr. T. Popa” University of Medicine and Pharmacy Iaşi, University Hospital “St. Spiridon” Iaşi, 1 Bulevardul Independentei, 700111 Iaşi, Romania; ^3^Department of General Surgery, University Hospital “Bagdasar-Arseni”, Şoseaua Berceni 10-12, 41914 Bucharest, Romania

## Abstract

*Background*. Pancreaticoduodenectomy is the potentially curative treatment for malignant and several benign conditions of the pancreatic head and periampullary region. While performing pancreaticoduodenectomy, early neck division may be impossible or inadequate in case of hepatic artery anatomic variants, suspected involvement of the superior mesenteric vessels, intraductal papillary mucinous neoplasm, and pancreatic head bleeding pseudoaneurysm. Our work aims to highlight a particular hind right approach pancreaticoduodenectomy in selected indications and assess the preliminary results. *Methods*. We describe our early hind right approach to the retropancreatic vasculature during pancreaticoduodenectomy by mesopancreas dissection before any pancreatic or digestive transection. *Results*. We used this approach in 52 patients. Thirty-two had hepatic artery anatomic variant and 2 had bleeding pancreatic head pseudoaneurysm. The hepatic artery variant was preserved in all cases out of 2 in which arterial reconstruction was performed. In nine patients with intraductal papillary mucinous neoplasms the pancreaticoduodenectomy was extended to the body in 6 and totalized in 3 patients. Seven patients with adenocarcinoma involving the portomesenteric axis required venous resection and reconstruction. *Conclusions*. Early hind right approach is advocated in selected cases of pancreaticoduodenectomy to improve locoregional vascular control and determine, safely and early, whether there is mesopancreas involvement.

## 1. Introduction

Pancreaticoduodenectomy (PD) is the treatment of choice for malignant and several benign conditions of the pancreatic head and periampullary region [1–4]. Since the first PD performed by Whipple in 1937, more than 70 technical improvements have been made, mainly related to pylorus preservation or reconstruction of pancreatico-digestive continuity and much less regarding the type of resection [1, 5–7]. Standard PD is usually performed with transection of the pancreatic neck before the superior mesenteric artery (SMA) dissection [8–10]. However, since the limited involvement of portomesenteric vein is no longer considered unresectable disease, the resectability is now assessed by whether or not the SMA is involved [11]. Moreover, the extended indications of PD in case of tumors associating hepatic artery (HA) variants or invading the mesentericoportal axis (borderline resectable pancreatic head adenocarcinomas) [11], as well as the importance to achieve R_0_ posteromedial resection margins (in adenocarcinomas and main duct-intraductal papillary mucinous neoplasms-MD-IPMN) [12], led to the development of so called “artery first” approaches [13]. Notable amongst these is an early right posterior approach to the superior mesenteric vessels, with mesopancreas (MP) dissection close to the origin of the SMA. The aim is to assess the resectability before taking an irreversible step, and variants of the arterial blood supply to the liver, to undertake the mobilization of the specimen before pancreatic or digestive division, and, if necessary, the safe venous clamping [5, 13–26].

We have adopted this hind right approach to the SMA since 2007 and have been using it combined with the early isolation and dissection free of the superior mesenteric vein (SMV) beneath the pancreas, as our “standard approach” PD in selected indications such as HA anatomic variants, suspected involvement of mesentericoportal axis or SMA, MD-IPMN, and pancreatic head bleeding pseudoaneurysm. It is very suitable to early assess the infiltration of SMV and SMA, allowing appropriate handling at the initial stages of the resection itself.

Although we have previously reported this approach related to PD in case of HA variants [5, 6], whereas we extended the indications, we describe how, when, and why to perform this modified right retropancreatic vascular approach PD and our current experience.

## 2. Methods

One hundred fifty consecutive patients have been registered for PD for benign and malignant diseases of the periampullary and pancreatic head region between January 1, 2007 and February 28, 2014. Among them, 52 (30 males and 22 females, median age 56.7 years; range 40–78 years) underwent PD with early hind right dissection. Patient characteristics are presented in Table 1. In 32 patients, the preoperative multidetector computed tomography revealed HA anatomic variants (Table 1): aberrant right HA (RHA) and replaced common HA (RCHA), with retropancreatic (28 cases) or intrapancreatic (4 cases) and retroportal course. Seven patients with adenocarcinoma had the added involvement of the portomesenteric vein. Nine patients with MD-IPMT were preoperatively assessed by abdominal multidetector computed tomography and endoscopic ultrasound with guided fine needle aspiration biopsy. A bleeding pancreatic head pseudoaneurysm was disclosed by computed tomography in 2 patients.

The procedures were performed by the same trained surgical team.

## 3. Surgical Technique

**Figure 1:**
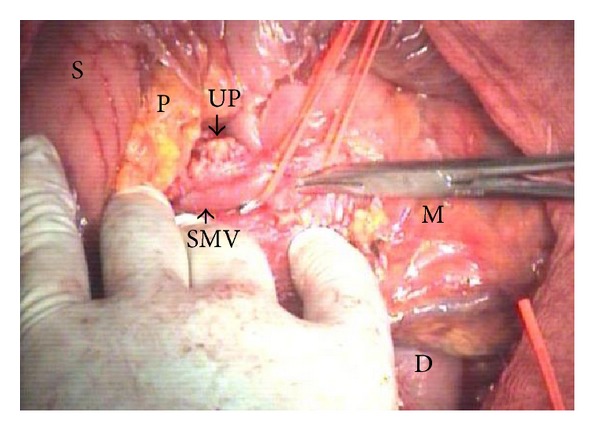
Early exposure of the SMV beneath the pancreas, on the anterior third duodenum; the vein is dissected free from the pancreas and uncinate process. SMV: superior mesenteric vein; D: duodenum; M: mesentery; P: pancreas; S: stomach; UP: uncinate process.

**Figure 2:**
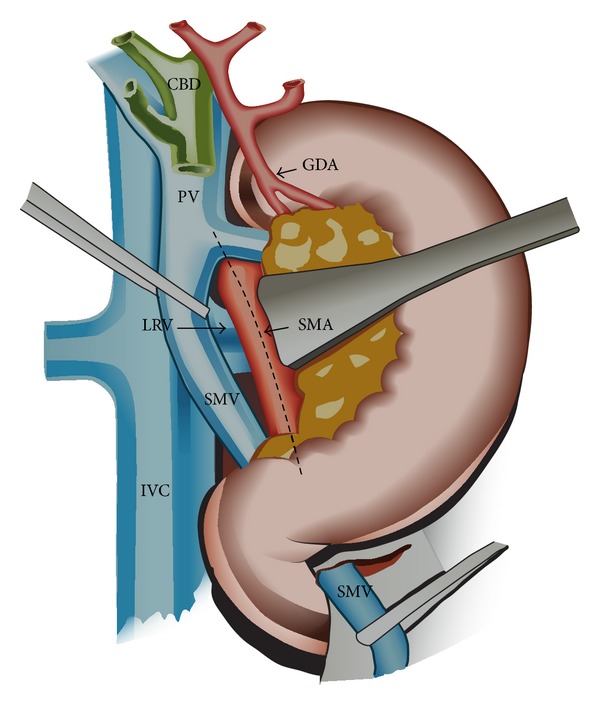
Hind right approach PD: after extended Kocher maneuver the duodenopancreas is retracted upwards and medially. Early posterior approach to the SMA (dissection of the MP along the discontinuous line) combined with an early approach to the SMV below the pancreas, where it passes over the third duodenum (the SMV is dissected free from the pancreas and uncinate, the right gastroepiploic and inferior pancreaticoduodenal veins are ligated, and a tunnel is created between the portomesenteric vein and pancreas towards the hepatic pedicle). CBD: common bile duct; GDA: gastroduodenal artery; IVC: inferior vena cava; LRV: left renal vein; P: pancreas; PV: portal vein; SMA: superior mesenteric artery; SMV: superior mesenteric vein.

**Figure 3:**
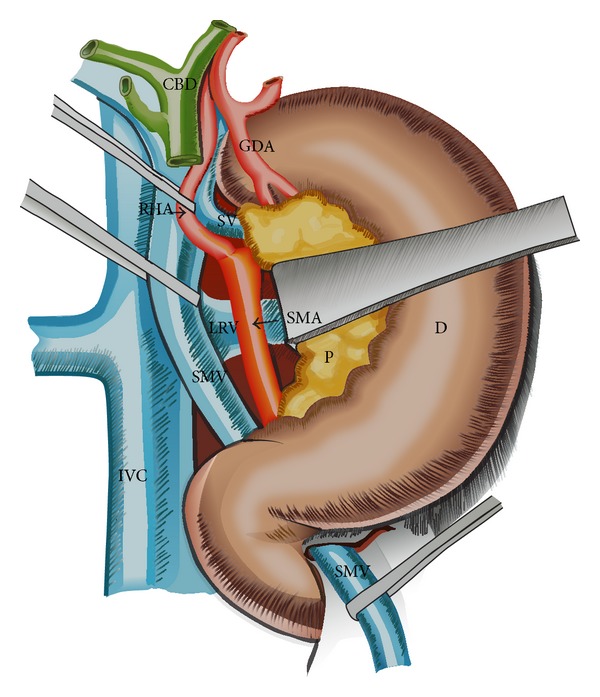
Hind right approach PD. Within the MP dissection, the SMA origin is detected in the angle between the left border of the IVC and the upper margin of the LRV. A RHA arising from the SMA is easily isolated 1-2 cm beneath SMA origin. The aberrant vessel is dissected upwards to the hepatic artery and preserved. The SMA is dissected along 4 cm towards the mesenteric root. CBD: common bile duct; D: duodenum; GDA: gastroduodenal artery; IVC: inferior vena cava; LRV: left renal vein; P: pancreas; SV: splenic vein; SMA: superior mesenteric artery; SMV: superior mesenteric vein.

The pancreas head is exposed by an extended Kocher maneuver carried out beyond the aorta, incision of the attachment of the transverse mesocolon to the right Gerota fascia, and opening of the lesser sac by separating greater omentum and transverse colon using a Liga-Sure device. The superior mesenteric vein (SMV) is early isolated below the pancreas, where it passes over the third duodenum and is dissected free from the pancreas and uncinate process (Figures 1 and 2), with ligation of the right gastroepiploic and inferior pancreaticoduodenal veins and early creation of a tunnel between pancreas and portomesenteric axis towards the hepatic pedicle (Figures 2 and 3). This step early detects whether or not the portomesenteric vein is involved and assesses the infiltration status of the infrapancreatic SMA. Behind the pancreas, the dissection must surpass the aorta to get full posterior leftwards mobilization of the duodenopancreas, and the plane between the SMV and the SMA is identified as part of the MP (Figures 2 and 3).

**Figure 4:**
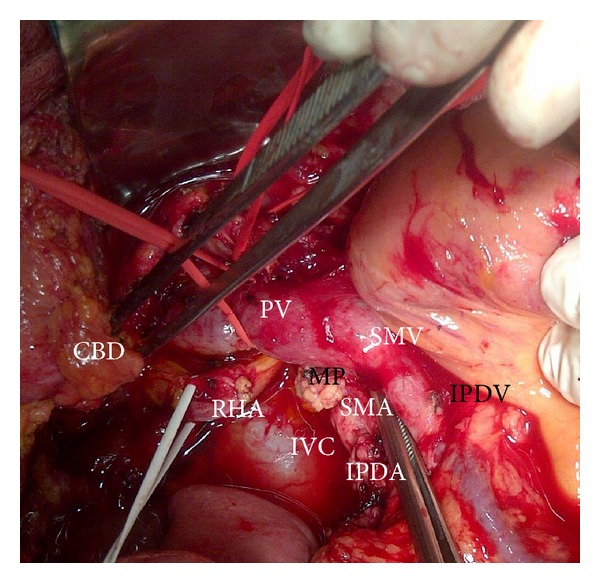
Hind right exposure of the MP and superior mesenteric vessels; the duodenopancreas with the tumor is retracted upwards and to the left; CBD: transected common bile duct; IVC: inferior vena cava; MP: dissected mesopancreas, between SMA origin and celiac trunk; PV: portal vein; RHA: right hepatic artery arising from the celiac trunk and retroportal path; SMA: superior mesenteric artery; SMV: superior mesenteric vein; IPDV: ligated inferior pancreaticoduodenal vein; IPDA: ligated inferior pancreaticoduodenal artery.

The retropancreatic dissection is carried on downwards from the inferior border of the Winslow foramen along the Treitz fascia, exposing the inferior vena cava on its left side, the upper margin of the left renal vein, and, in between, the origin of the SMA along with the posterior pancreatic capsule (Figures 2, 3, and 4).

**Figure 5:**
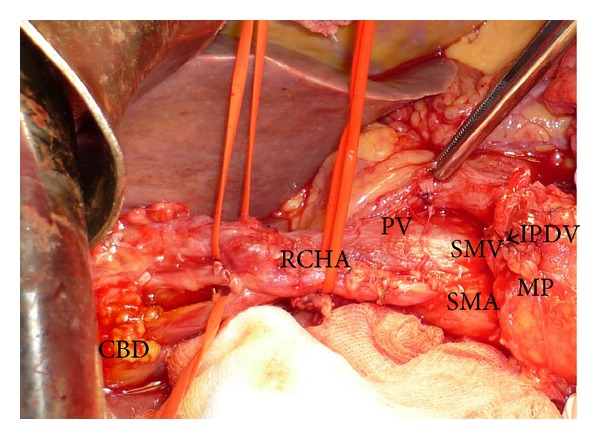
Hind right dissection of the SMA with resected MP and spared intrapancreatic path RCHA, just before the digestive and pancreatic transection; CBD: common bile duct; MP: mesopancreas; PV: portal vein; RCHA: replaced common hepatic artery with intrapancreatic path and early bifurcation in right and left hepatic branches; SMA: superior mesenteric artery; SMV: superior mesenteric vein; IPDV: ligated inferior pancreaticoduodenal vein.

The SMA origin is identified in this angle, and along its adventitial plane the MP (including RPL or retropancreatic medial margin) is dissected and removed “step-by-step.” The MP is inserted on its right aspect, in a frontal plane behind the pancreas (Figures 2, 3, and 4). This dissection is pursued over 3-4 cm, from the SMA origin until its entrance into the mesentery, using progressive exposure and gentle medial retraction of the portal vein (PV), which is also freed from the MP (Figures 3 and 4). The superior and inferior pancreaticoduodenal arteries are identified and ligated (Figure 4). The MP is retracted to the right and all lymphatic and perineural tissue between SMA and SMV is removed to achieve negative resection margins. Possible SMA invasion can be early detected, due to MP involvement, avoiding the risk of nonradical resection. Complete excision of the connective tissue between the origin of the SMA and the (CT) is performed as well. This exposure enables the dissection of a RHA originating from SMA or CT (Figure 3) or a RCHA arising from the SMA (Figures 4 and 5). The vessel usually arising 1-2 cm from the SMA origin is looped and freed from the MP, upwards to the hepatic pedicle (Figures 3, 4, and 5). Its safeguarding is generally possible. To facilitate the SMA and aberrant RHA or RCHA dissection, the duodenopancreas is retracted en bloc upwards, ventrally and to the left. Limited dissection along the right side of the SMA is advocated (Figures 2, 3, and 4), to avoid extensive removal of the perivascular nervous plexus, resulting in postoperative intestinal motility troubles.

The hepatic pedicle is approached after this extended dissection. The cholecystectomy is performed, the common and proper HAs are isolated, and the right gastric vessels and gastroduodenal artery are identified and clamped to make sure that the arterial flow either in hepatic or gastric arteries remains normal and there is no unrecognized CT stenosis. The gastroduodenal artery is divided, as well as the common bile duct above the entry of the cystic duct (Figures 3 and 4). This improves the exposure of the suprapancreatic PV. During periportal lymphadenectomy one should be aware of an eventual accessory or replaced RHA originating from the SMA or CT, or to a RCHA from the SMA. If present, this vessel running upwards behind the PV is looped (Figures 3 and 4). The SMV is entirely dissected at the inferior pancreatic margin with ligation of all veins draining the uncinate process, which is exposed up to the right side of the SMA. At this stage the posterior wall of the portomesenteric axis is entirely exposed. The Treitz ligament is divided, allowing mobilization of the duodenojejunal junction, so the specimen to be removed reaches the right side of the mesenteric root.

Once the radicality of PDR is established jejunal and distal gastric division are undertaken according to Whipple procedure using stapling devices. The last step of the resection is the pancreatic neck transection, just in front of the PV using a usual “cold” scalpel. When pancreatic division must be deviated towards the body, the dorsal pancreatic artery and collaterals of both the SMA and the SMV (from the lower edge of the pancreas) are divided. In case of involvement of the portomesenteric confluence, the splenic vein is controlled behind the body. Adequate mobilization of the mesentery and right colon is necessary to perform safely “en bloc” resection and venous reconstruction. This mobilization is useful in case of limited portomesenteric invasion, in order to avoid vein grafting during venous reconstruction.

In case of IPMN extending from the head to the body, the retropancreatic mobilization is done leftwards and the splenic vessels are dissected with successive ligation of their collaterals. When the pancreatic body is mobilized sufficiently the pancreas can be divided at any level, or entirely removed. Frozen section analysis is performed at the sectioned champs, to assess the malignant status of the remnant pancreas. Reconstruction phase, drainage, and postoperative care are similar to those from standard PD. During the operative procedure we use standard dissection and ligatures, monopolar section and coagulation. The Liga-Sure device is used during Kocher maneuver and division of the lesser and greater omentum.

## 4. Results

HA variants were intraoperatively confirmed in all 32 cases. The aberrant vessel was preserved in 30 cases. A RCHA originating from the SMA was involved by an enlarged lymph nodes mass behind the pancreatic head (borderline resectable pancreatic head cancer) in 2 patients, so a segmental resection of the involved RCHA had to be performed with arterial reconstruction, using the reversed splenic artery in both cases. Right hind approach PD was also performed in emergency in two cases of pancreatic head bleeding pseudoaneurysm, with early ligation of the pseudoaneurysm feeding artery, originating from the inferior pancreaticoduodenal artery. Seven patients with borderline resectable ductal adenocarcinoma involving the portomesenteric confluence required en bloc resection, mobilization of the right colon, and mesentery root followed by mesentericoportal veno-venous suture. When vascular reconstruction was required, clamping time did not exceed 22 minutes. Anastomotic patency and normal blood flow were confirmed by Doppler ultrasound at the end of the procedure.

The same approach was used in 9 patients with MD-IPMN (6 PD extended to the body-IPMN in the head, uncinate, or neck and 3 total PD-IPMN diffusely involving the main pancreatic duct).

Since we routinely perform this approach in selected indications, no conversion to standard PD was undertaken.

The median operative time was 295 minutes (range 225–435) and median blood loss was 760 mL (range 215–1090). The short-term outcome related to this approach is shown in Table 2. For the malignant tumors, a R_0_ resection was achieved in 32 patients and a R_1_ resection in 5 patients (14%), (all with borderline resectable pancreatic head cancers). No R_2_ resection was noted. The follow-up has lasted until patient death or until the cut-off date of February 28, 2014. The median follow-up time was 32.5 months (range 6.5–72). At the time of the last follow-up, 39 patients were still alive. If only patients with pancreatic cancer were taken into account, median survival time was 19.1 months (range 8.5–32).

## 5. Discussion

Because of continuous decrease in mortality rate, PD is nowadays routinely performed for tumors of the pancreatic head and periampullary region, with or without invasion of the mesentericoportal axis and even in IPMN. The early hind approach to the MP during PD on the right side of the SMA, before the digestive and pancreatic continuity that should be interrupted, is of particular interest in case of HA abnormality, with RHA originating from SMA or CT, or RCHA from the SMA, suspected involvement of the SMA, MD-IPMN extended from the pancreatic head to the body, and involvement of the portomesenteric axis. Recently we performed in emergency this hind right approach Whipple in two patients with bleeding pancreatic head pseudoaneurysm. In this setting, early ligation of the inferior pancreaticoduodenal artery (feeding the bleeding pancreatic head pseudoaneurysm) enabled a steady hemostasis and removal of the lesion. From the technical variants described as “artery first” approach to PD [13], we routinely adopted a right posterior approach to the SMA, additionally combined with early isolation and dissection free of the SMV beneath the pancreas (Figures 1, 2, and 3). It has become the standard practice in our unit in the above mentioned indications. The potential advantage of this approach is that technical difficulties, which may be encountered either due to tumor infiltration of the SMA, SMV, or the main PV, can be clearly assessed and handled appropriately at the initial stages of the resection itself. It gathers the advantages of a posterior “artery first” approach PD with a modified uncinate process first approach, regarding the following:

1. identification of SMA involvement either at the origin or at uncinate;
2. identification of the portomesenteric vein involvement requiring en bloc resection;
3. identification and preservation of HA variants;
4. adequate retropancreatic lymphadenectomy;
5. minimal bleeding by early ligation of the IPDA and IPDV;
6. effectiveness in obesity, postchemotherapy status, and peripancreatic inflammation;
7. mobilization of the whole gland before transection;
8. removal of large tumors of the pancreatic head extending to the uncinate.

Standard PD implies the creation of a tunnel between the pancreatic neck and the PV, followed by neck transection so the pancreatic continuity is interrupted before radicality of the resection can be assessed. The late determination of the MP infiltration status means that the surgeon is already committed to resection. Even in some recent series, nonradical PD is presented [27, 28]. Moreover, in the standard PD, dissection of a RHA or RCHA is usually performed late, when bleeding from the resection specimen decreases the exposure of the SMA and of an aberrant RHA. Early neck transection is also not suitable when the pancreatic neck and/or the portomesenteric axis are involved [18, 19] or in MD-IPMN extended to the body or diffusely affecting the pancreas [12, 16, 17]. One of the difficulties of PD lies in the variability of peripancreatic vascular anatomy. Preoperative assessment of variant pattern of the arterial blood supply to the liver (variants, strictures) is necessary to avoid unnecessary complications, such as fatal hepatic injury [29, 30]. Accidental ligation of HA may result in hepatic necrosis, ischemic biliary tract injury, or anastomotic complications [31, 32]. Moreover, injury of an aberrant HA during PD relates to a breakdown of bilioenteric anastomosis, because the blood supply to the cranial part of the common bile duct is entirely dependent on the RHA after PD [32–34]. RHA or RCHA from SMA may be situated behind or within the pancreas head or along its ventral side [35–37]. We could not confirm its course before dissecting and isolating it from the SMA origin within the MP dissection. In our series, in the vast majority of patients with HA anatomic variant, the aberrant vessel was spared. In two patients this artery was willingly sacrificed for oncological reasons, but reconstructed.

Pancreatic head carcinoma with venous limited involvement can be safely resected with a long-term survival similar to that observed after radical resection without venous involvement [13, 18, 23, 38, 39]. In such situation, the best option is to perform “en bloc” venous resection in order to obtain R_0_ resection [11]. By using the hind right approach, after transection of the pancreatic isthmus, the tumor remains attached only to the involved veins, so clamping of the portomesenteric confluence is easier and shorter [39, 40]. Mobilization of the right colon and mesentery root are useful to avoid vein grafting during reconstruction of the PV [41]. Since the pancreatic transection is performed at the end, congestion and bleeding are less likely whereas the venous drainage of both the specimen and bowel are compromised minimally during most of the procedure [20, 25]. Moreover, there is a reduced intraoperative blood loss, due to an early ligation of the inferior pancreaticoduodenal artery [20, 40].

In MD-IPMN, the most frequent localization is the pancreatic head, but involvement of the body may occur [12, 16, 17]. In this setting and particularly in malignancies [12], en bloc resection requires pancreatic division of the body rather than the neck. Early hind right approach to the MP facilitates pancreatic mobilization towards the left. By the early approach to the SMV beneath the pancreas, our technique enables the total pancreatectomy as mobilization can be achieved without transecting gland. In fact, final transection of the pancreas can be performed at the desired place if it is enough separated from the splenic vessels, preventing tumor opening, which might disseminate cancer into the abdomen. Furthermore, dissection along the splenic vessels can be extended up to the splenic hilum allowing splenic preservation if the whole pancreas must be resected [12, 16, 17].

Removal of all small vessels, nerves, and lymphatic nodes and networks within the retroperitoneal adipose tissue, the so-called “Total Mesopancreas Excision,” increases the rate of negative resection margins, thus reducing the local recurrence rate and improving the survival [20, 21, 42]. The MP is the retroperitoneal thin soft tissue, within retropancreatic attachments comprising connective tissue, perivascular nervous plexus, and lymph nodes belonging to posterior pancreaticoduodenal vessels. The term MP seems to replace or rather include the classical term of retroportal lamina (RPL), retroperitoneal/posteromedial margin, or retropancreatic capsule, which has a frontal disposition between the pancreas and the SMA [10, 14, 15, 20, 21]. The MP is of great interest with respect to curative resection in malignancies since it is the primary site for R_1_ resection [21, 43]. In our series, the positive resection margins-R_1_ (5 patients) were associated to borderline resectable pancreatic head cancers and extended to uncinate, but the rate (14%) is lower than that reported in the literature for similar cases (18–24%) [11, 44]. MP dissection remains one of the most challenging steps in PD no matter what type of approach is used (standard, posterior, or artery first below the pancreas). At present, there is no evidence based on large series concerning the benefits of the “MP first” or “artery first” approaches over the standard PD [45]. A drawback of our study is the heterogeneity of the indications and pathology conditions for PD (malignant/benign diseases, IPMN). Therefore, a comparative study with a matching cohort of patients undergoing standard PD should be difficult. As a matter of fact, this was not an end-point of our study, but to highlight the advantages of our approach in selected indications of PD. Further prospective randomized studies are necessary to assess the real clinical impact of the MP excision in achieving negative resection margins, decreasing local recurrence, and improving the long-term survival of patients resected for pancreatic cancer.

It is worth noting that a limitation of the retropancreatic approach PD was reported in obese patients and those with extensive peripancreatic inflammation [26]. Nevertheless, the surgeon should face the same concern in standard PD too. We encountered this problem in our patients with chronic pancreatitis and those with previous chemotherapy. These conditions render the procedure more difficult rather during vascular isolation and dissection. However, since our approach early exposes the retropancreatic vasculature and dissects the portomesenteric vein below the pancreas and uncinate, it comes to be useful even in peripancreatic inflammation, as it facilitates retropancreatic tunneling above PV and whole pancreas mobilization before transection.

In conclusion, early hind right dissection combined with early exposure of the SMV below the pancreas is a useful technique to expose the retro- and infrapancreatic mesenteric vasculature early during PD. Because of its advantages, we use it routinely in patients with HA anatomic variants, suspected SMA involvement, limited invasion of the mesentericoportal axis, MD-IPMN, and bleeding pancreatic head pseudoaneurysm. MP first dissection facilitates the radicality and safety of PD and enables early vascular control. Further prospective studies are required to assess its advantages over standard PD, since there is no consensus worldwide.

## Figures and Tables

**Table 1:** Patient characteristics.

Characteristics	Number	%
Patients	52	
Median age (ys)	56.7	
Males/females	30/22	
ASA classification		
I	12	23%
II	33	63%
III/IV	7	14%
Diseases (pathologic entity)		
Malignant disease		
Total	**35**	**68%**
Pancreatic ADK	23	44%
Ampullary ADK	3	6%
Distal CBP ADK	6	12%
Duodenal ADK	2	4%
Neuroendocrine pancreatic tumours	1	2%
Benign Disease		
Total	**8**	**16%**
Insulinoma	3	6%
Chronic pancreatitis	5	10%
IMPT	**9**	**18%**
Hepatic artery anatomic variant		
Total	**32**	**61%**
(1) Aberrant right hepatic artery (RHA)	**24**	**47%**
Origin		
From the SMA	21	40%
From the CT	3	6%
Type		
Replaced RHA	17	32%
Accessory RHA	7	13%
(2) Aberrant common hepatic Artery (CHA)	**8**	16%
Origin		
From the SMA	7	13%
From the aorta	1	2%
Type		
Replaced CHA	8	16%

**Table 2:** Short-term outcome after pancreaticoduodenectomy with early hind right dissection.

Surgical complications (27 events in 22 patients) (42%)	
Pancreaticojejunostomy leak	7 (13%)
Remnant pancreas acute pancreatitis	2 (4%)
Delayed gastric emptying	9 (17%)
Pancreatic stump hemorrhage	2 (4%)
Hemorrhage from gastric stapled suture	2 (2%)
Wound infection	5 (10%)
Relaparotomy	3 (6%)
Hospital mortality	2 (4%)
Median hospital stay (days)	16
